# Effects of a Combined Plyometric and Resistance Training Programme on Adolescent Rugby Union Players

**DOI:** 10.3390/muscles4020017

**Published:** 2025-06-02

**Authors:** Cian M. Walsh, Joseph I. Esformes, Jeremy A. Moody, Paul J. Byrne

**Affiliations:** 1 Department of Health and Sport Sciences, South East Technological University (Kilkenny Road Campus), R93 V960 Carlow, Ireland; cianowalsh@gmail.com; 2 Cardiff School of Sport and Health Sciences, Cardiff Metropolitan University, Cardiff CF5 2YB, UK; jesformes@cardiffmet.ac.uk (J.I.E.);; 3 School of Physical Education and Sports, Nisantasi University, Istanbul 34398, Turkey

**Keywords:** plyometric exercise, weight training, youth, team sport, jump training, muscle contraction, countermovement jump

## Abstract

Background: Previous work has found that combining plyometric and resistance training in a single session can be effective in improving maximum strength (muscle contraction ability), vertical jumping, and sprint acceleration performance in young soccer players. However, the literature is scarce in relation to young rugby union players. Thus, the purposes of the present study were to examine the effects of a 6-week combined training (CT) programme that combined plyometric and resistance training in the same session on physical performance measures in adolescent male rugby union players and whether a 4-week period of plyometric training exclusion (Detraining) affects training adaptations. Methods: The participants (*n* = 15) completed a 6-week CT intervention and 4 weeks of plyometric detraining during the schoolboy rugby union in-season. A performance testing battery was conducted pre-intervention, post-intervention, and 4 weeks post-intervention. Results: A repeated measures ANOVA showed a significant effect for time on CMJ variables (mean power, mean force, and modified reactive strength index [RSI]), 3RM back squat strength, and 505 test time (*p* < 0.05). Pairwise comparisons revealed that CMJ variables (mean force, mean power, and peak power), 3RM back squat strength, and 505 test performance significantly increased from pre-intervention to post-intervention (*p* < 0.05). The 4-week plyometric detraining period significantly augmented CMJ variables (mean force, mean power, and modified RSI) and performance measures (standing broad jump [SBJ], 20 m sprint time, 505 test, and 3RM strength). Conclusions: The findings indicate that a 6-week CT programme can augment measures of lower-body power, maximal strength, and change of direction performance in adolescent male rugby union players, and a 4-week resistance training-only period (plyometric detraining) does not negatively affect performance adaptations.

## 1. Introduction

Rugby union is a team sport characterised by various intermittent activities, with high-intensity activity interspersed with periods of low physical exertion [1]. Rugby players require endurance, strength, speed, change of direction (COD) ability, and power for in-game activities, including tackling, sprinting, evading, and jumping [2]. Forwards are typically engaged in high-intensity activity when competing for the ball in rucks, lineouts, and scrums, while backs perform more high-intensity running [3]. While differences in positional fitness requirements exist in rugby union, all players are expected to possess a wide array of physical capabilities due to the intermittent and chaotic nature of the sport. Therefore, strength and conditioning practices are standard in preparing rugby players of various playing levels and ages for competition [4].

Resistance training is typically performed by athletes seeking improvements in muscular fitness, such as muscular strength, hypertrophy, power, and sport-specific adaptations [5]. Suchomel et al. [6] suggest that muscular strength adaptations resulting from dynamic resistance training have greater carry-over to dynamic exercise performance than other resistance training methods. In addition to dynamic resistance training, plyometric training can play a pertinent role in athletic performance enhancement. As Suchomel et al. [6] noted, plyometric training typically involves a rapid stretch-shortening cycle (SSC) element, where a previous eccentric muscle contraction augments a concentric muscle contraction. The potential transfer of muscular strength to power can make plyometric training a valuable component of training programmes, particularly in sporting contexts [6]. According to Newton and Kraemer [7], combining resistance with plyometric training allows the force and velocity components of maximum power to be trained concurrently. Additionally, research has suggested that a combination of resistance training and plyometrics is favourable for maximum strength development compared to a single training mode [8,9]. In the present study, “combined training” (CT) refers to performing plyometric and resistance training in the same session where plyometric training is completed first, followed by resistance training in the form of strength training using compound exercises with free weights in a single session [10].

To date, only one study has examined the effects of CT on the physical performance of rugby union players [11]. The authors explored the effects of a 4-week combined rugby conditioning and plyometrics programme (*n* = 19) compared to a non-plyometric rugby conditioning programme (*n* = 16) on physical performance in university-aged male rugby union players. Notably, the results of the study indicated that the CT group improved significantly more than the control group in 20 m sprint speed, Wingate anaerobic test performance, and COD performance (*p* < 0.05) [11]. Studies on CT in male soccer players [10,12,13,14] and court-based athletes [15,16] have shown significant increases in lower-body muscular strength and jump performance, respectively. When focusing on soccer players, a study on U-20 male elite soccer players compared three different CT approaches within a session over an 8-week period [10]. The findings showed that CT with plyometrics performed before strength training induced significant gains in maximum strength and vertical jumping performance. However, 10 and 20 m sprint speed significantly decreased. In a second study on soccer players, Zghal et al. [14] compared 7 weeks of an active control (soccer training programme) to combined resistance training and plyometric and sprint training to plyometric and sprint training only in youth club-level male players. The authors suggested that combined resistance training and plyometric and sprint training was the approach to use to enhance maximum strength, jump, and sprint performance. The following study considered the impact on court-based players, where CT was compared to plyometric training alone on youth male volleyball players over a 16-week period [16]. The CT was conducted by performing the strength exercise prior to the plyometric exercise. CT led to significant improvements in vertical jumping, 5 and 10 m sprint performance, medicine ball throw, and lower-body flexibility.

Although the performance benefits of CT for team sports athletes have been explored, there is a lack of studies examining the effects of a plyometric detraining period following either CT or plyometric training in athletes. Fathi et al. [16] found that measures of sprint, jump, and power performance following a CT programme decreased significantly (*p* < 0.05) during 16 weeks of plyometric detraining in youth volleyball players. In contrast, Santos and Janeira [17] observed no significant decrease in vertical jump measures following a plyometric detraining period of 16 weeks in adolescent male basketball players. However, it remains unclear whether a detraining period would affect previous adaptations from CT in field sport settings such as rugby union.

In adolescent team sports, training programmes must maximise performance while being time-efficient due to limited training time and other commitments, including schoolwork. Therefore, the complementary nature of CT may efficiently enhance multiple physical qualities in team sports athletes. However, whether CT benefits adolescent rugby union players and whether training adaptations remain during detraining is yet to be established. Therefore, the aims of the present study were (1) to investigate the effects of a 6-week CT training programme on physical performance measures in adolescent male rugby union players and (2) to examine if a 4-week period of resistance training only (plyometric detraining) impacts training adaptations. It was hypothesised that physical performance would significantly improve following a CT training programme in adolescent male rugby union players and that a detraining period would negatively impact training adaptations.

## 2. Results

### 2.1. Test-Retest Reliability

The ICC ranges were 0.567–0.915 for all CMJ variables and 0.763–0.93 for all remaining performance variables. The CMJ variables showed moderate to very high intra-rater reliability, while all other variables had high to very high reliability across multiple measurements [18].

### 2.2. Countermovement Jump

**Figure 1:**
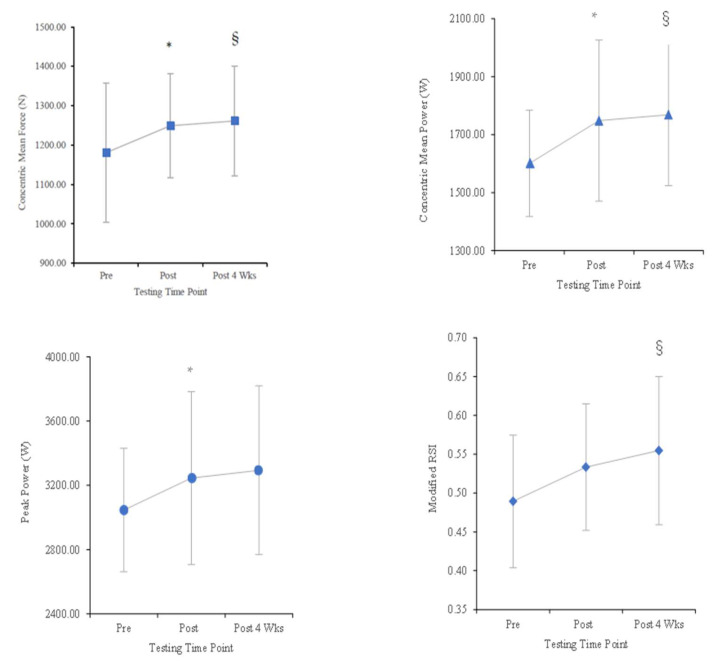
Countermovement jump concentric mean force, concentric mean power, peak power, and modified RSI (mean ± SD) for pre-, post-, and 4 weeks post-combined training intervention. * *p* < 0.05 difference between pre-intervention and post-intervention. § *p* < 0.05 difference between pre-intervention and 4 weeks post-intervention.

The repeated measures ANOVA showed a significant effect of time on concentric mean force (F = 7.66, df: 1.19, 16.75, *p* = 0.010, partial eta: 0.354), concentric mean power (F = 6.43, df: 2, 28, *p* = 0.005, partial eta: 0.315), and modified RSI (F = 3.487, df: 2, 28, *p* = 0.044, partial eta: 0.199). No significant differences between time points were revealed for CMJ height, concentric peak velocity, or concentric RFD (Table 1). Pairwise comparisons revealed significant increases from pre- to post-intervention for concentric mean force (*p* = 0.016, ∆ = 5.8%, d = 0.71), peak power (*p* = 0.027, ∆ = 6.5%, d = 0.64), and concentric mean power (*p* = 0.007, ∆ = 9.2%, d = 0.81), with no difference between post-intervention and 4 weeks post-intervention time points (Table 1 and Figure 1). Significant increases were also observed from pre-intervention to 4 weeks post-intervention time points in concentric mean force (*p* = 0.011, ∆ = 6.8%, d = 0.75), concentric mean power (*p* = 0.008, ∆ = 10.4%, d = 0.80), and modified RSI (*p* = 0.040, ∆ = 13.4%, d = 0.59; Table 1 and Figure 1). A non-significant but practical SWC was found between pre- and post-intervention for concentric peak velocity (*p* = 0.321, SWC = 0.06, ∆ = 0.07 (2.6%), d = 0.27), concentric RFD (*p* = 0.363, SWC = 147.8, ∆ = 245.4 (23.4%), d = 0.24), and modified RSI (*p* = 0.139, SWC = 0.02, ∆ = 0.04 (9.0%), d = 0.40).

### 2.3. Standing Broad Jump

**Figure 2:**
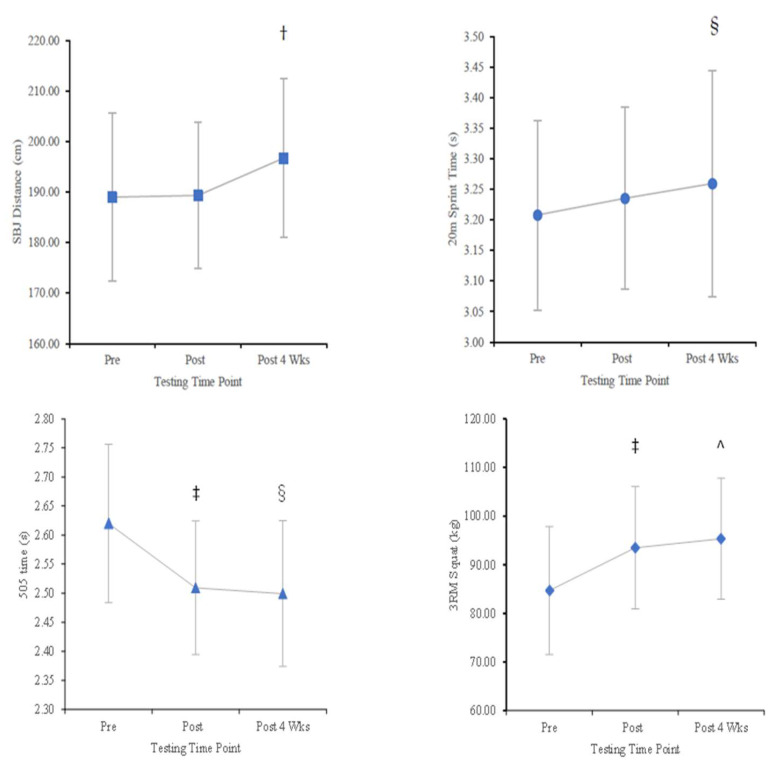
SBJ distance, 20 m sprint time, 505 time, and 3RM squat (mean ± SD) for pre-, post-, and 4 weeks post-combined training intervention. ‡ *p* < 0.001 difference between pre-intervention and post-intervention. ^ *p* < 0.001 difference between pre-intervention and 4 weeks post-intervention. § *p* < 0.05 difference between pre-intervention and 4 weeks post-intervention. † *p* < 0.05 difference between post-intervention and 4 weeks post-intervention.

A non-significant, small to moderate effect was found between pre-intervention and 4 weeks post-intervention values (*p* = 0.112, SWC = 3.33, ∆ = 7.7 (4.1%), d = 0.44). A paired t-test showed a significant increase (*p* = 0.041, ∆ = 3.9%, d = 0.58) from post-intervention to 4 weeks post-intervention (Table 2 and Figure 2).

### 2.4. Speed

A paired t-test revealed a significant increase (*p* = 0.030, ∆ = 1.60%, d = 0.62) in 20 m sprint time from pre-intervention to 4 weeks post-intervention (Table 2 and Figure 2).

### 2.5. Change of Direction

A repeated measures ANOVA showed a significant effect for time was evident for 505 times (F = 13.53, df: 2, 28, *p* < 0.001, partial eta: 0.491). Pairwise comparisons indicated that 505 times significantly decreased from pre- to post-intervention (*p* < 0.001, ∆ = −4.2%, d = 1.22) and from pre-intervention to 4 weeks post-intervention (*p* = 0.002, ∆ = −4.6%, d = 0.96) (Table 2 and Figure 2).

### 2.6. Three-Repetition Maximum Back Squat

A repeated measures ANOVA showed a significant time effect for the 3RM load (F = 19.09, df: 1.23, 17.23, *p* < 0.001, partial eta: 0.577). Pairwise comparisons displayed that 3RM load significantly increased from pre- to post-intervention (*p* < 0.001, ∆ = 10.4%, d = 1.15) and from pre-intervention to 4 weeks post-intervention (*p* < 0.001, ∆ = 12.6%, d = 1.18) (Table 2 and Figure 2).

## 3. Discussion

The purposes of the present study were to examine the effects of a 6-week combined training (CT) programme that combined resistance and plyometric training in the same session on physical performance measures in adolescent male rugby union players and whether a 4-week period of plyometric training exclusion (Detraining) affects training adaptations. CMJ performance, COD performance, and 3RM back squat strength significantly improved from pre- to post-intervention, showing moderate to large effect sizes (*d* = 0.64–1.22). Additionally, a period of plyometric detraining induced a significant improvement in SBJ performance. However, there were non-significant differences for CMJ measures, sprint performance (5 and 20 m), COD, and 3RM back squat strength. The only previous study for direct comparison with the present study was conducted by Pienaar and Coetzee [11], who examined the effects of a 4-week CT programme in university-aged male rugby union players. The authors reported significant increases in Wingate test power and COD performance post-intervention. The comparable improvements in lower-body power and COD observed in the present study may be due to the similarities in the study design, participant characteristics, and sample sizes in each study. Although CMJ height was unaffected by the CT intervention, the significant increases observed in concentric mean force, concentric mean power, and peak power pre- to post-training suggest that lower-body force and power production may be positively impacted in the present study. This concurs with Adams et al. [19], who compared the efficacy of barbell back squat, plyometric, and combined squat and plyometric training programmes on lower-body power production over 6 weeks in resistance-trained university-aged males. Their main finding was that CT improved vertical jump performance significantly more than the squat-only or plyometric-only training groups. Notably, their participants were untrained in plyometrics, like ours. When comparing the present study’s findings to previous studies using CT in soccer players, the present study is in agreement with Zghal et al. [14] in relation to maximum strength. However, the present study showed vertical jump height and sprint performance over 5 and 20 m to be unchanged. These contrasting results may be due to Zghal et al. [14] including sprint training with the plyometric exercises and because the plyometric jump intensity and volume were higher than in the present study. Furthermore, the present study compares favourably for maximum strength with a study on U-20 male elite soccer players where three different CT approaches were compared within a session over an 8-week period [10]. Nonetheless, sprint and vertical jump performance in the present study remained unchanged, whereas Kobal et al. [10] reported a significant decrease in sprint speed and a significant increase in vertical jump height. In relation to the sprint outcomes in the present study and this study, the difference may be due to the order effect of plyometric training and resistance training. Previous research has shown a significant improvement in sprint performance when plyometric exercises are performed following resistance training [20]. In terms of vertical jump performance, reasons for the contrasting results may be due to the present study using a 6-week period and drop jumps from a 30 cm box, whereas Kobal et al. [12] ran the study for 8 weeks and progressed the drop jump box height from 30 to 45 cm. Moreover, it has been previously suggested that the specific jump that is to be tested is also included in the training in order for participants to transfer inter-muscular co-ordination from the training to the specific jump [21]. The present study shows contrasting vertical jump and sprint performance results compared to a study in which CT was compared to plyometric training alone in youth male volleyball players over a 16-week period [16]. The differing results are possibly due to our study lasting 6 weeks versus this study, which was conducted for 16 weeks. Moreover, this study progressed the CT group over the 16-week duration in relation to the hurdle and drop jump drop heights for the plyometric exercises.

According to de Villarreal et al. [9], individuals with no plyometric training experience will likely respond favourably to training initially due to nervous system adaptations, including increased motor unit recruitment, synchronisation, excitability, and neural drive. Therefore, the novel stimulus of plyometric training may have resulted in neuromuscular adaptations, which may explain the improvements observed in the participants’ lower-body power production. The present study has shown that CT may enhance maximal strength and COD performance [9,11,22]. When comparing CT to plyometric training for maximal strength development, de Villarreal et al. [9] found that CT produced significantly greater strength increases (ES = 1.21) than plyometric training (ES = 0.64). The improved intermuscular coordination and mechanical characteristics of the muscle tendon complex of the plantar flexors, resulting from adding plyometric training, are possible mechanisms for the 3RM back squat strength enhancement observed in the present study [23]. Increased motor unit recruitment, power output, and rapid force production following plyometric training may be responsible for COD performance enhancement [22,24]. Therefore, the neural adaptations and observed lower-body power improvements following CT may have been the primary mechanisms that promoted COD performance enhancement.

Including a plyometric detraining period provided further insight into the efficacy of CT in adolescent rugby union players. Several measures (CMJ mean force and mean power, COD, 3RM back squat strength) that significantly improved from pre- to post-intervention were positively affected following 4 weeks of plyometric detraining, with these measures experiencing significant improvements from pre- to 4 weeks post-intervention. Moreover, modified RSI from the CMJ and the SBJ experienced a significant improvement from pre-CT training to 4 weeks post-plyometric detraining. However, 20 m sprint performance was significantly attenuated from pre-intervention to 4 weeks post-plyometric detraining. The resistance and rugby union training during the detraining period likely contributed to enhancing the benefits obtained during the CT intervention, and the additional 4-week period of resistance and rugby union training led to enhancements in modified RSI and SBJ. The negative effect of attenuated 20 m sprint performance was due to the lack of sprint training over a 20 m distance. According to a review, to improve sprint performance over distances up to 20 m, distance-specific sprint training is required [25]. The continued pursuit of maximal strength gains in the participants’ resistance training programme was vital due to the correlation between muscular strength and power and sports skill performance, such as jumping, sprinting, and COD [26]. Thus, the continuation of resistance training during the plyometric detraining period induced significant gains in certain CMJ measures, COD, 3RM back squat strength, and SBJ, which supports the relationship between maximal strength and these athletic qualities. In addition, the specificity of sprinting and changing direction during rugby union training may have complemented the maintenance of 505 test performance following plyometric detraining.

Previous research on detraining periods following combined or plyometric training interventions has produced equivocal findings. Santos and Janeira [17] examined the effects of a 16-week plyometric detraining period following a 10-week plyometric training programme during the in-season in adolescent male basketball players. The authors observed no significant decreases across multiple jump performance tests following detraining [17]. However, it was challenging to define the post-intervention period in their study as plyometric detraining due to the nature of basketball, which may have stimulated the maintenance of previously acquired plyometric adaptations. Research by Fathi et al. [16] explored the effect of a 16-week CT or plyometric training programme followed by a detraining period of equal duration in male youth volleyball players. The results showed that sprint, power, and jump performance measures returned to pre-intervention values following 16 weeks of detraining [16]. The 16-week detraining period in the above studies is far greater than the 4-week timeframe used in the present study. Therefore, while in our study, performance measures were enhanced following CT, they were unaffected by plyometric detraining. A more extended detraining period, e.g., 12–16 weeks, may have allowed for stronger inferences on the effect of plyometric detraining on athletic performance.

Although the present findings suggest improvements in lower-body power and strength, these results should be interpreted cautiously, given the small sample size (*n* = 15) and absence of a control group. Further research with larger cohorts and matched controls is required to substantiate these observations. Recruiting participants for the study proved challenging due to commitments to multiple sports and schoolwork. Additionally, the study occurred during the in-season period of the schoolboy rugby union season. Therefore, during CT or testing sessions, participants may have been fatigued due to rugby union training and matches. The 4-week period of detraining being shorter than the 6-week intervention also limits the applicability of the study to similar research with more extended periods of plyometric detraining that match or exceed the intervention in duration [16,17].

## 4. Materials and Methods

### 4.1. Experimental Design

**Figure 3:**
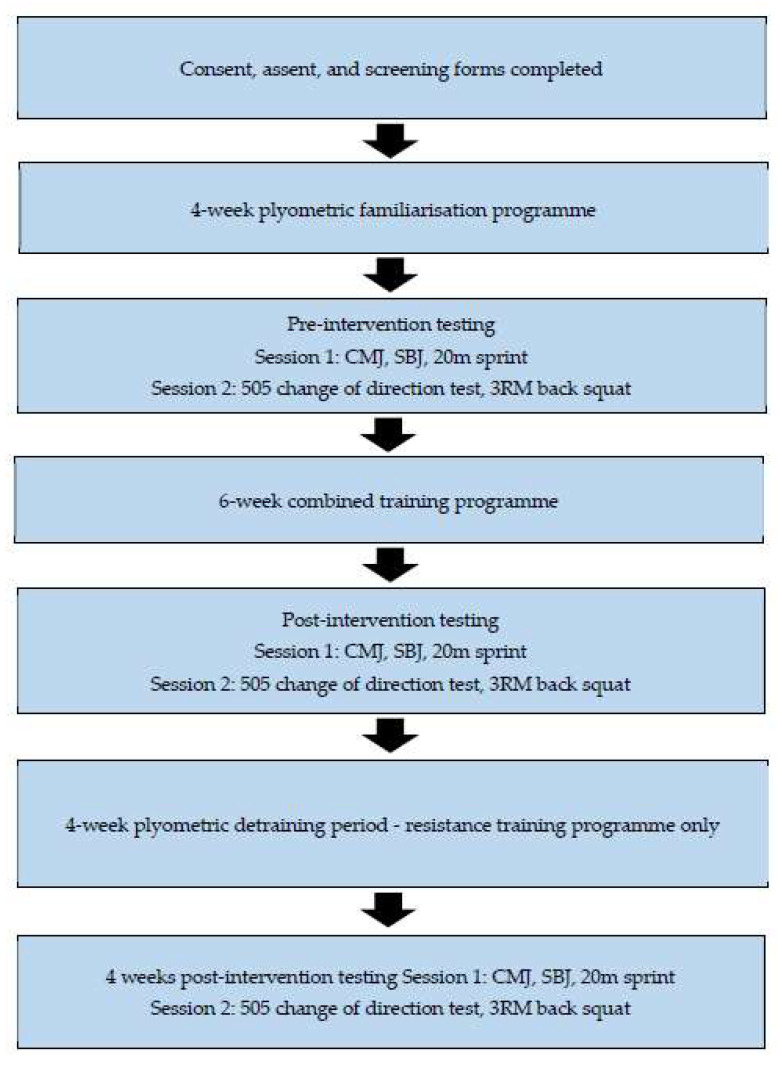
Flow chart of the experimental design of the study. CMJ = countermovement jump, SBJ = standing broad jump, 3RM = three-repetition maximum.

The current study used a repeated measures design, with testing occurring on three separate occasions: pre- and post-six weeks of plyometric and resistance training and a second post-test following four weeks of plyometric detraining while maintaining resistance training (Figure 3). All participants completed the intervention.

### 4.2. Participants

Adolescent male rugby union players (*n* = 15) (age: 16.8 ± 0.5 years; height: 176.6 ± 4.2 cm; body mass: 69.8 ± 5.9 kg; BMI: 22.3 + 1.6 kg/m^2^) from St. Mary’s College secondary school, Co. Dublin, senior development rugby squad were recruited to participate in the present study. Participants were approached to volunteer for the study via a presentation of the study design and aims and the associated benefits and risks involved with participation. Participant information forms were provided to further clarify the details of the study. As the participants were younger than 18 years of age, parental consent forms and participant assent forms were also completed before the study. A minimum of 6 months of resistance training and no plyometric training experience were necessary for inclusion in the study. Applicants could not partake in the current study if they had a musculoskeletal injury in the previous three months that would impact their ability to complete the training intervention, which was determined through medical screening.

### 4.3. Training Protocols

#### 4.3.1. Plyometric Training for Familiarisation

As the participants had no previous experience with plyometric training, all participants completed a low-intensity familiarisation plyometric training programme lasting 4 weeks before initiating the CT intervention, consisting of low-intensity jumping and hopping exercises that provided a foundation for the participant’s landing mechanics, improved technique, and reduced the risk of injury during the study (Table 3). Furthermore, the participants performed resistance training in addition to the plyometric training for familiarisation. The participants were instructed to perform the plyometric exercises shown in Table 3 with maximal effort. After the familiarisation plyometric training programme, the CT protocol was implemented over a 6-week in-season. Plyometric training occurred before each resistance training session started, aiming to improve lower-body power production and use of the SSC (Table 4).

#### 4.3.2. Plyometric Training Sessions

Vertical, horizontal, and lateral plyometric exercises (Table 4) were implemented in each session to target performance adaptations in different movement directions [27,28,29]. Drop jumps were performed using a 30 cm box to drop and a 50 cm box for the subsequent box jump. The participants were instructed to spend as little time on the ground as possible during drop jumps to encourage short ground contact times and rapid force production [27,29]. Training session volume was increased by five ground contacts biweekly, and the plyometric exercises selected were moderate- to high-intensity [27,29,30]. Exercise intensity was increased by progressing exercises to more advanced variations biweekly. Rest times during single-repetition exercises were set at 15 s between each repetition and 30 s between each set, with 60 s of rest between the sets for repeated-repetition exercises. Rest intervals between exercises were 60 s long [11,31].

#### 4.3.3. Resistance Training

All participants completed the same resistance training programme (Table 5), designed and supervised by a qualified strength and conditioning coach from the school, and performed twice weekly, lasting no longer than one hour. The resistance training programme was followed for six weeks as part of the CT programme and was continued during the four-week plyometric detraining period. The programme consisted of free-weight compound exercises to promote muscular strength and hypertrophy adaptations. Each exercise was performed at 70–85% of the participants’ one-repetition maximum (1RM) for 3–4 sets of 6–12 repetitions [32]. Participants who were unable to perform body mass chin-ups performed band-assisted chin-ups. Rest periods between sets were 2–3 min [33]. The participants were instructed to perform the eccentric phase of the exercise over 2 s for each repetition. Rating of perceived exertion (RPE) values were used on selected exercises to prescribe exercise intensity. The RPE scale (1–10) has been validated as a practical method of assigning individualised intensity due to the inconvenience of 1RM testing [34].

### 4.4. Testing Procedures

The participants underwent six days of testing, including 2 days separated by 48 h at each time point: pre-intervention, post-intervention, and 4 weeks post-intervention. The first day of testing involved performing the countermovement jump (CMJ), standing broad jump (SBJ), and 20 m sprint test. The second testing day was 48 h later, consisting of lower-body maximal strength and COD testing. The participants were familiarised with the testing protocols 1 week before the pre-testing began. All jumping and maximal strength testing was completed in the school’s gym, while sprint and COD testing was performed indoors in the school’s sports hall.

#### 4.4.1. Countermovement Jump Test

The CMJ is a highly reliable (a = 0.98) and valid (r = 0.87) test for measuring lower-body explosive power in active males [35]. The CMJ test was performed using the ForceDecks FDMax dual plate system (VALD, Brisbane, Australia) with a sampling frequency of 1000 Hz per plate. The participants were instructed to stand with their feet shoulder-width apart with one foot on each of the dual force plates and to keep their hands on their hips throughout the jump. Once the set-up was complete, the participants rapidly dropped to a height of their preference by flexing their knees and hips and then exploded upwards, jumping as high as possible. The participants completed three jump trials with 2 min between trials. The highest jump was used for data analysis. The CMJ variables recorded for analysis and their definitions can be found in Table 6.

#### 4.4.2. Standing Broad Jump Test

The SBJ was measured using a fibreglass tape measure and a start line. The participants began by standing with their feet hip-width apart behind the start line. The participants were instructed to jump forward as explosively as possible and to stick the landing on both feet. The distance between the start line and the back of the heel closest to the start line was recorded as the test score. The participants completed three trials with 2 min of rest between the trials. The furthest jump was recorded for data analysis [37].

#### 4.4.3. 20 m Sprint Test

Speed was assessed through maximal sprinting trials and measured using timing gates (Dashr Motion Performance, Omaha, NE, USA). Timing gates were set at the start line, at 5 m and 20 m. The participants set up 0.5 m behind the start gates in a two-point split stance. They were instructed to sprint as fast as possible through the gates at 20 m before slowing down for two trials, with a minimum rest of 3 min between sprints. The time values (s) of the two trials were recorded [29]. The lowest time of the two trials of the 20 m sprint times and the 5 m sprint time were used for data analysis.

#### 4.4.4. Three-Repetition Maximum Back Squat Test

Lower-body maximal strength was assessed with the three-repetition maximum (3RM) barbell back squat (kg). The 3RM barbell back squat was performed using squat racks, barbells, and weight plates from the school gym (Fitness Equipment Ireland, Dublin, Ireland). The participants completed three warm-up sets with low- to moderate-intensity loads on the barbell back squat [38]. Previous 3RM testing data were used to estimate a 3RM load for the first trial. If a participant was successful with this trial, a load was added until the participant could not complete three repetitions [39]. Squat depth was set as the thighs parallel to the floor at the bottom of the squat [38]. Safety pins were used in all squat racks, and a qualified strength and conditioning coach was a spotter during all maximal trials. The highest 3RM load for each participant was recorded for data analysis [38].

#### 4.4.5. Change of Direction Test

COD performance was measured using the 505 test. One set of timing gates was placed 5 m from a turning line. The starting line was 10 m from the timing gates and 15 m from the turning point. The participants were instructed to sprint as fast as possible through the timing gates, turn 180 degrees at the turning line on their stronger foot, and run back through the timing gates as quickly as possible. The rest interval between the trials was 2 min. In line with the speed test, the lowest of the two trials was recorded [40].

### 4.5. Statistical Analyses

Statistical analysis was performed using SPSS for Mac Software (version 28, IBM SPSS, Chicago, IL, USA). All dependent variables are presented as mean ± standard deviation (SD). Data were analysed for normality using skewness and kurtosis values, central tendency on Q-Q normality plots and histograms, and 5% trimmed mean values. A one-way repeated measures ANOVA was conducted for each dependent variable to measure the differences in test scores across each time point (pre, post, 4 weeks post). Intraclass correlation coefficient (ICC) values were obtained to assess intra-rater reliability for each dependent variable. A Bonferroni post hoc adjustment was used to compare and interpret the significant values observed. Statistical significance was set at *p* ≤ 0.05. The smallest worthwhile change (SWC) was calculated as 0.2 × between-subject SD of the baseline test. Effect sizes were reported as Cohen’s *d* values, with 0.2 = small effect, 0.5 = moderate effect, and 0.8 = large effect [41].

## 5. Conclusions and Practical Applications

In conclusion, the present study’s findings showed that a 6-week CT programme may have enhanced lower-body power, maximal strength, and COD performance in adolescent male rugby union players, while 4 weeks of plyometric detraining did not affect these performance gains. This could indicate that performance may have continued to improve if plyometric training had been maintained, as gains were observed during the initial 6-week period of CT and plateaued during the subsequent resistance-only phase. However, given that adaptations occur to a greater extent early in the training period and in the absence of a comparison group, further studies are required to clarify the specific contributions of CT when performed in isolation compared to CT. The adaptations during this CT period from plyometric training were likely due to neuromuscular adaptations, including enhanced neural drive, motor unit recruitment, excitability, synchronisation, intermuscular coordination, and mechanical characteristics of the muscle tendon complex of the plantar flexors [9,23]. Nevertheless, based upon our findings, CT may be applied to adolescent male rugby union players who desire these performance adaptations and seek enhancements in the maximum strength and power necessary for meeting the demands of rugby union. Additionally, the time efficiency of CT suggests that it could be particularly beneficial in adolescent and schoolboy rugby union settings. The ability to train multiple fitness components in the same training session can be valuable to adolescent athletes, with reduced training opportunities due to school commitments. The present study builds upon previous research on CT and plyometric detraining while extending our current understanding of CT to the adolescent male rugby union population. Future research investigating CT and plyometric detraining in adolescent male rugby union players should be conducted with larger sample sizes, control groups, and longer detraining periods to reinforce the use of CT for enhancing physical performance.

## Figures and Tables

**Table 1:** Countermovement jump measures (mean ± SD) for pre-, post-, and 4 weeks post-combined training intervention.

	Pre	Post	Post 4 Weeks
Jump height (cm)	32.9 ± 3.4	33.1 ± 3.4	32.5 ± 1.9
Concentric peak velocity (m/s)	2.5 ± 0.2	2.6 ± 0.2	2.67 ± 0.2
Concentric mean force (N)	1180 ± 176	1248 ± 132 *	1261 ± 139 §
Peak power (W)	3046 ± 384	3244 ± 537 *	3293 ± 525
Concentric mean power (W)	1601 ± 183	1748 ± 277 *	1768 ± 244 §
Concentric RFD (N/s)	1048 ± 739	1293 ± 925	1119 ± 753
Modified RSI (m/s)	0.49 ± 0.09	0.53 ± 0.08	0.55 ± 0.10 §

RFD = rate of force development; RSI = reactive strength index. * *p* < 0.05 difference between pre-intervention and post-intervention. § *p* < 0.05 difference between pre-intervention and 4 weeks post-intervention.

**Table 2:** Performance measures (mean ± SD) for pre-, post-, and 4 weeks post-combined training intervention.

	Pre	Post	Post 4 Weeks
SBJ distance (cm)	189.0 ± 16.6	189.3 ± 14.4	196.7 ± 15.6 †
Sprint 5 m (s)	1.11 ± 0.08	1.11 ± 0.07	1.11 ± 0.10
Sprint 20 m (s)	3.21 ± 0.16	3.24 ± 0.15	3.26 ± 0.18 §
505 (s)	2.62 ± 0.14	2.51 ± 0.11 ‡	2.50 ± 0.13 §
3RM squat (kg)	84.6 ± 13.1	93.5 ± 12.5 ‡	95.3 ± 12.4 ^

SBJ = standing broad jump; 3RM = three-repetition maximum. ‡ *p* < 0.001 difference between pre-intervention and post-intervention. ^ *p* < 0.001 difference between pre-intervention and 4 weeks post-intervention. § *p* < 0.05 difference between pre-intervention and 4 weeks post-intervention. † *p* < 0.05 difference between post-intervention and 4 weeks post-intervention.

**Table 3:** Plyometric training familiarisation programme displaying the sets and repetitions for the respective exercises across a 4-week period.

	Exercise	Sets × Repetitions
Week 1	Snap-downs	2 × 5
	Drop and land (30 cm box)	2 × 5
	Box jump (30 cm box)	1 × 5
	Standing broad jump	1 × 5
Week 2	Ankle hops	2 × 8
	Lateral hop and hold	2 × 6
	Double broad jump	3 × 2
Weeks 3–4	Countermovement jump	2 × 3
	Pogo hops	2 × 5
	Triple broad jumps	2 × 3
	Lateral ankle hops	2 × 6

**Table 4:** Plyometric training programme that includes the warm-up exercises and the respective exercises conducted with vertical training, followed by horizontal training and lateral training, with their training volume across the 6-week period.

	Vertical Training	Sets × Reps	Horizontal Training	Sets × Reps	Lateral Training	Sets × Reps	Total Session CTs	Total Weekly CTs
Warm-up	CMJ	1 × 3	Standing broad jump	1 × 3	Lateral ankle hops	1 × 5		
Weeks 1–2	Repeated CMJ	3 × 3	Repeated broad jumps	2 × 4	Lateral cone hops	2 × 6	45	90
	Pogo jumps	2 × 8						
Weeks 3–4	Double-leg tuck jumps	2 × 8	Single-leg zig-zag cone hops	2 × 5	Lateral hurdle jumps	2 × 7	50	100
	Drop jumps	2 × 5						
Weeks 5–6	Hurdle jumps	3 × 5	Alternate-leg bounding	2 × 6	Lateral bounding	2 × 8	55	110
	Drop jumps	3 × 4						

CMJ = countermovement jump, CTs = bilateral feet ground contacts.

**Table 5:** Resistance training programme displaying the sets, repetitions, and intensity for the respective exercises.

**Resistance Training Session 1**		
**Exercise**	**Sets** × **Repetitions**	**Intensity**
Barbell bench press	4 × 6	80% 1RM
Barbell Romanian deadlift	4 × 6	80% 1RM
Dumbbell goblet squat	3 × 10	RPE 8-9
Dumbbell row	3 × 10	RPE 8-9
Banded dead bug	3 × 8	RPE 8-9
**Resistance Training Session 2**		
**Exercise**	**Sets** × **Repetitions**	**Intensity**
Barbell front squat	4 × 6	80% 1RM
Dumbbell floor press	3 × 10	RPE 8-9
Dumbbell Romanian deadlift	3 × 10	RPE 8-9
Chin-ups	3 × 8	RPE 8-9
Bear crawl arm raise	3 × 8	RPE 8-9

**Table 6:** Definitions of countermovement jump variables (adapted from Heishman et al. [36]).

CMJ Variable	Definition
CMJ height (flight time) (cm)	Maximal jump height calculated using flight time
Concentric peak velocity (m/s)	Highest velocity achieved during the concentric phase
Concentric mean force (N)	Mean force during the concentric phase
Peak power (W)	Greatest power achieved
Concentric mean power (W)	Mean power during the concentric phase
Concentric RFD (N/s)	RFD from the start of the concentric phase to peak power
Modified RSI (m/s)	Jump height (flight time) divided by contraction time

## Data Availability

The original contributions presented in this study are included in the article. Further inquiries can be directed to the corresponding author.

## Abbreviations

The following abbreviations are used in this manuscript:

3RM	Three repetition maximum
CMJ	Countermovement jump
COD	Change of direction
CT	Combined training
ICC	Intraclass correlation coefficient
RFD	Rate of force development
RPE	Rate of perceived exertion
RSI	Reactive strength index
SBJ	Standing broad Jump
SSC	Stretch-shortening cycle
SWC	Smallest worthwhile change
